# Favourable neurological outcome following paediatric out-of-hospital cardiac arrest: a retrospective observational study

**DOI:** 10.1186/s13049-023-01165-y

**Published:** 2023-12-21

**Authors:** Alexander Fuchs, Deliah Bockemuehl, Sabrina Jegerlehner, Christian P. Both, Evelien Cools, Thomas Riva, Roland Albrecht, Robert Greif, Martin Mueller, Urs Pietsch

**Affiliations:** 1 Department of Anaesthesiology and Pain Medicine, Bern University Hospital, Inselspital, University of Bern, Freiburgstrasse, Bern, 3010, +41 31 664 14 65 Switzerland; 2 Unit for Research in Anaesthesia, Department of Paediatric Anaesthesia, IRCCS, Istituto Giannina Gaslini, Genova, Italy; 3 Swiss Air-Ambulance (Rega), Zurich, Switzerland; 4 Department of perioperative Intensive Care Medicine, Cantonal Hospital St. Gallen, St. Gallen, Switzerland; 5 Department of Emergency Medicine, Bern University Hospital, Inselspital, University of Bern, Bern, Switzerland; 6 Department of Anaesthesiology, Children’s Hospital Zurich, Zurich, Switzerland; 7 Unit for Anaesthesiological Investigations, Department of Anaesthesiology, Pharmacology, Intensive Care and Emergency Medicine, Geneva University Hospitals and University of Geneva, Geneva, Switzerland; 8 University of Bern, Bern, Switzerland; 9 School of Medicine, Sigmund Freud University Vienna, Vienna, Austria; 10 European Resuscitation Council (ERC) Research NET, Niel, Belgium

**Keywords:** Out-of-hospital Cardiac Arrest, Children, HEMS, Chain-of-survival, Resuscitation, Advanced life support

## Abstract

**Background:**

Out-of-hospital cardiac arrest (OHCA) in children is rare and can potentially result in severe neurological impairment. Our study aimed to identify characteristics of and factors associated with favourable neurological outcome following the resuscitation of children by the Swiss helicopter emergency medical service.

**Materials and methods:**

This retrospective observational study screened the Swiss Air-Ambulance electronic database from 01-01-2011 to 31-12-2021. We included all primary missions for patients ≤ 16 years with OHCA. The primary outcome was favourable neurological outcome after 30 days (cerebral performance categories (CPC) 1 and 2). Multivariable linear regression identified potential factors associated with favourable outcome (odd ratio – OR).

**Results:**

Having screened 110,331 missions, we identified 296 children with OHCA, which we included in the analysis. Patients were 5.0 [1.0; 12.0] years old and 61.5% (n = 182) male. More than two-thirds had a non-traumatic OHCA (67.2%, n = 199), while 32.8% (n = 97) had a traumatic OHCA. Thirty days after the event, 24.0% (n = 71) of patients were alive, 18.9% (n = 56) with a favourable neurological outcome (CPC 1 n = 46, CPC 2 n = 10). Bystander cardiopulmonary resuscitation (OR 10.34; 95%CI 2.29–51.42; *p* = 0.002) and non-traumatic aetiology (OR 11.07 2.38–51.42; *p* = 0.002) were the factors most strongly associated with favourable outcome. Factors associated with an unfavourable neurological outcome were initial asystole (OR 0.12; 95%CI 0.04–0.39; *p* < 0.001), administration of adrenaline (OR 0.14; 95%CI 0.05–0.39; *p* < 0.001) and ongoing chest compression at HEMS arrival (OR 0.17; 95%CI 0.04–0.65; *p* = 0.010).

**Conclusion:**

In this study, 18.9% of paediatric OHCA patients survived with a favourable neurologic outcome 30 days after treatment by the Swiss helicopter emergency medical service. Immediate bystander cardiopulmonary resuscitation and non-traumatic OHCA aetiology were the factors most strongly associated with a favourable neurological outcome. These results underline the importance of effective bystander and first-responder rescue as the foundation for subsequent professional treatment of children in cardiac arrest.

**Supplementary Information:**

The online version contains supplementary material available at 10.1186/s13049-023-01165-y.

## Introduction

Out-of-hospital cardiac arrest (OHCA) is a leading cause of death in Europe and must be treated immediately to ensure survival [1]. Paediatric OHCA is a rare event, with incidences varying between 3.5 and 4.2 per 100,000 in European countries [2, 3]. OHCA can be broadly subdivided into traumatic and medical (non-cardiac and cardiac) causes. In addition to immediate bystander cardiopulmonary resuscitation (CPR), proper basic life support (BLS) by first responders and advanced life support (ALS) treatment by organized emergency medical services, patient outcomes can include severe neurological impairment or death. Poor survival rates in paediatric OHCA are reported, at around 15% for non-traumatic and only 5% for traumatic cardiac arrest. Unfortunately, data on survival rates often do not report favourable neurological outcomes, [4] which is the aim of every resuscitation attempt. Additionally, paediatric OHCA is a significant public health issue, as poor outcomes result in the loss of substantial numbers of years of life and cause profound distress for those affected (e.g. patients, families, healthcare teams).

The sparse existing literature describes risk factors associated with poor neurological outcomes for patients including being less than one year old and being male [5, 6].

Emergency responses to paediatric OHCA differ widely between countries and regions. Switzerland has ground-based paramedic-staffed emergency medical systems and physician-staffed helicopter emergency medical systems (HEMS).

The aim of this study was to investigate survival and neurological outcome following cases of paediatric OHCA that were treated by the Swiss Air-Ambulance (Rega), the national HEMS. Furthermore, we aimed to describe the characteristics of the patients, as well as to identify the factors associated with a 30-day favourable neurological outcome.

## Materials and methods

### Study design

The Ethics Committee of Eastern Switzerland (EKOS 23/089, St. Gallen, Switzerland) approved this retrospective observational study, thus waiving the need for informed consent. The study was conducted in line with the Declaration of Helsinki and the Swiss Act on Human Research. Our reporting conforms to the applicable STROBE guidelines [7].

### Setting

Switzerland had in 2022 approximately 8.7 million inhabitants, with around 19% under the age of 20 years. In Switzerland, ground-based, paramedic-staffed emergency medical services and physician-staffed HEMS jointly provide pre-hospital emergency medicine, including ALS, that covers the whole country. Additionally, there are nationwide first-responder systems with voluntary persons (healthcare and non-healthcare professionals) providing BLS. First Responders are registered persons with specific training in BLS. The Swiss Air-Ambulance (Rega) is the largest national HEMS provider in Switzerland, operating from 12 bases with 16 helicopters and treating approximately 12,000 patients annually. A Rega helicopter crew comprises a pilot, a flight paramedic and an anaesthesiologist with additional pre-hospital emergency-medicine training.

### Patients

We screened the Rega HEMS electronic database from 01-01-2011 until 31-12-2021 (11 years) for patients with a National Advisory Committee for Aeronautics (NACA) [8] score of > 5 (NACA 6 = respiratory or cardiac arrest with need for CPR and ROSC; NACA 7 = death with or without CPR) and documented resuscitation variables (i.e. manual chest compressions, mechanical chest compression device, cardiopulmonary resuscitation, ICD-10 codes, text search), including all primary HEMS missions involving patients ≤ 16 years with an OHCA. We excluded missions with an NACA < 6, secondary missions (i.e. inter-hospital transfers) [9] and missions involving patients > 16 years of age. Furthermore, we excluded missions with missing data on the primary outcome and missions with insufficient documentation of the resuscitation in the HEMS protocol.

### Variables

We extracted all the mission data from the HEMS electronic database which contains medical and administrative data. The data included mission day and times (i.e. response time, on-scene time), helicopter hoist operation, remote area not accessible by car, patient age and sex, and activity prior to cardiac arrest. We then manually extracted the following data from the medical HEMS protocols: co-morbidity, resuscitation characteristics (i.e. aetiology of the cardiac arrest (traumatic vs. non-traumatic), dispatcher identified, dispatcher-assisted CPR, witnessed cardiac arrest, bystander CPR, first-responder BLS, first rhythm, interventions and medication during CPR, survival outcomes (i.e. Return of Spontaneous Circulation (ROSC), as well as on-scene survival and emergency department survival. Data on 30-day and 1-year survival and 30-day neurological outcome was obtained from hospital discharge letters and follow-up investigations. All data were transferred into an electronic research database following the Utstein-style template for cardiac arrest [10].

### Study outcomes

The primary outcome was defined as favourable neurological outcome after 30 days according to cerebral performance categories (CPC) 1 and 2 [11]. In contrast, an unfavourable neurological outcome was defined as CPC > 2 and death. Secondary outcomes were return of spontaneous circulation (ROSC), survival on-scene, survival at the emergency department and survival one year after the OHCA.

### Statistical methods

The statistical analysis was conducted using STATA® 16.1 (StataCorp, College Station, Texas, USA).

For categorical variables, the absolute number and percentage were reported. Continuous variables were presented with their mean and standard deviation (SD) or median and interquartile range (IQR), depending on normality testing using the Shapiro Wilk test. Also depending on normality testing, Student’s t-tests or Wilcoxon rank sum tests were used for continuous variables. In contrast, chi-squared tests or Fisher’s exact tests were used, as appropriate, for categorical variables in order to test for associations with favourable neurological outcomes after 30 days. Logistic univariable regression analyses were performed within the total cohort to assess the candidates for multivariable analysis. Factors that showed at least very weak evidence of an association with the outcome (*p* < 0.2) in the univariable analysis were included in a stepwise logistic regression model with backward selection (*p* < 0.2) to derive the final multivariable model. Only factors with no more than 4% of missing data in the total cohort were included in the multivariable model. The final model was applied to a subgroup analysis, excluding both patients without any resuscitation attempt (bystander CPR, BLS or ALS) (subgroup 1) and patients without any resuscitation attempt (bystander CPR, BLS or ALS) or with ROSC already achieved (subgroup 2). Odds ratios with 95% confidence interval (CI) were used as effect sizes. The area under the receiver operating characteristic curve (AUROC) was calculated in order to evaluate the discriminatory power and overall performance of the model. An AUROC value greater than 0.8 indicates an excellent fit, [12] while a *p*-value of < 0.05 was considered statistically significant.

A Sankey diagram (Statistical Software Components S459154, Boston College Department of Economics, MA, USA) was used to visually represent the patient’s course over a period of one year following cardiac arrest.

## Results

**Fig. 1:**
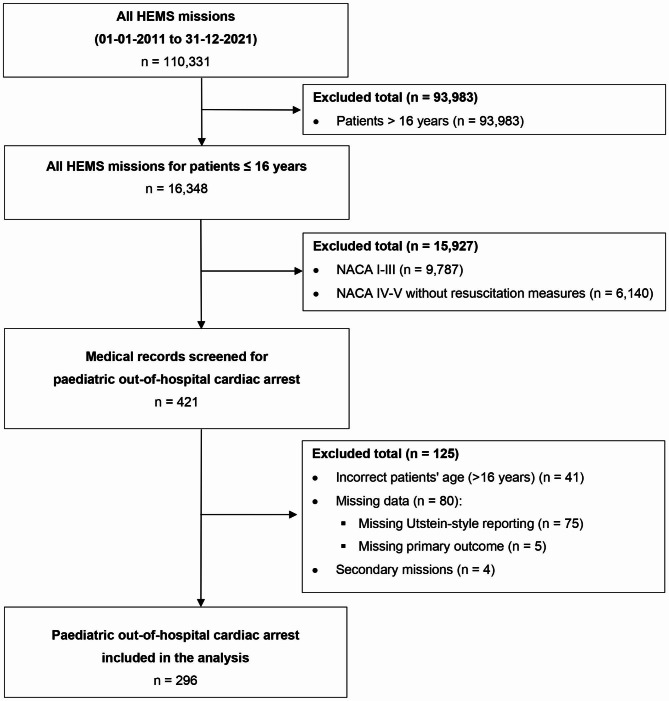
Study flow chart

We screened 110,331 missions in the Rega-HEMS electronic medical database, of which 296 met the inclusion criteria (Fig. 1).

Table 1 presents the baseline characteristics of the analysed cases that were related to favourable neurological outcome. Patients were 5.0 [1.0;12.0] years of age and 61.5% (n = 182) of them were male. More than two-thirds had a non-traumatic aetiology of the cardiac arrest (67.2%, n = 199), while 32.8% (n = 97) had a traumatic aetiology. The leading cause of cardiac arrest was hypoxia (36.5%, n = 108) and the most prevalent comorbidities were cardiovascular conditions (6.4%, n = 19). Cardiac arrest was witnessed in 36.8% (n = 109) of the cases, but 66.6% (n = 197) of patients in cardiac arrest received bystander CPR. The predominant initial rhythm recorded was asystole (56.1%, n = 167).

**Table 1:** Baseline characteristics of all cases and adjusted to 30-day favourable neurological outcome defined as cerebral performance categories (CPC) 1 and 2. Data presented in n (%) or median [Q1; Q2]

		Total	30d CPC 1/2		
	N	(n = 296)	Yes (n = 56)	No (n = 240)	*P*-value
MISSION & RESCUE CHARACTERISTICS					
Weekend mission	296	80 (27.0)	20 (35.7)	60 (25.0)	0.104
Season	296				
Winter		62 (20.9)	12 (21.4)	50 (20.8)	
Spring		85 (28.7)	15 (26.8)	70 (29.2)	
Summer		94 (31.8)	21 (37.5)	73 (30.4)	
Fall		55 (18.6)	8 (14.3)	47 (19.6)	0.682
Helicopter hoist operation	296	15 (5.1)	1 (1.8)	14 (5.8)	0.214
Remote location	296	51 (17.2)	10 (17.9)	41 (17.1)	0.890
Response time, minutes	280	16.0 [13.0; 21.0]	15.0 [11.0; 18.0]	17.0 [13.0; 22.0]	< 0.001
On-scene time, minutes	238	33.5 [24.0; 51.0]	26.0 [18.0; 33.0]	37.0 [27.0; 57.0]	< 0.001
PATIENT CHARACTERISTICS					
Age, years	296	5.0 [1.0; 12.0]	3.5 [1.0; 8.5]	6.0 [1.0; 13.0]	0.152
Age group	296				
< 1 year		56 (18.9)	11 (19.6)	45 (18.8)	
1–6 years		114 (38.5)	28 (50.0)	86 (35.8)	
7–16 years		126 (42.6)	17 (30.4)	109 (45.4)	0.091
Sex, male		182 (61.5)	33 (58.9)	149 (62.1)	0.662
COMORBIDITIES					
Known comorbidity	296	62 (20.9)	11 (19.6)	51 (21.2)	0.790
Cardiovascular	296	19 (6.4)	4 (7.1)	15 (6.2)	0.806
Respiratory	296	11 (3.7)	1 (1.8)	10 (4.2)	0.396
Neurological	296	11 (3.7)	3 (5.4)	8 (3.3)	0.471
Syndrome	296	7 (2.4)	1 (1.8)	6 (2.5)	0.751
Premature birth	296	4 (1.4)	1 (1.8)	3 (1.2)	0.755
Diabetes	296	1 (0.3)	0 (0.0)	1 (0.4)	0.628
Other	296	9 (3.0)	1 (1.8)	8 (3.3)	0.544
CPR CHARACTERISTICS					
Aetiology	296				
Traumatic cardiac arrest		97 (32.8)	3 (5.4)	94 (39.2)	
Non-traumatic cardiac arrest		199 (67.2)	53 (94.6)	146 (60.8)	< 0.001
Dispatcher-identified cardiac arrest	296	148 (50.0)	32 (57.1)	116 (48.3)	0.235
Dispatcher-assisted CPR	296	44 (14.9)	8 (14.3)	36 (15.0)	0.892
Witnessed cardiac arrest	296	109 (36.8)	27 (48.2)	82 (34.2)	0.050
Bystander CPR	296	197 (66.6)	52 (92.2)	145 (60.4)	< 0.001
Resuscitation delay > 2 min	296	157 (53.0)	12 (21.4)	145 (60.4)	< 0.001
ALS before HEMS arrival	296	206 (69.6)	36 (64.3)	170 (70.8)	0.337
Tracheal tube	296	193 (65.2)	22 (39.3)	171 (71.2)	< 0.001
Body temperature, Celsius°	76	33.5 [30.9; 35.0]	33.8 [32.2; 35.7]	33.0 [30.5; 34.6]	0.118
Resuscitation attempted	296				
Yes		245 (82.8)	32 (57.1)	213 (88.8)	
No		51 (17.2)	24 (42.9)	27 (11.2)	< 0.001
Chest compressions	296	241 (81.4)	29 (51.8)	212 (88.3)	< 0.001
First rhythm	296				
Asystole		155 (52.4)	4 (7.1)	151 (62.9)	
Pulseless Electrical Activity		45 (15.2)	5 (8.9)	40 (16.7)	
Pulseless Ventricular Tachycardia		1 (0.3)	0 (0.0)	1 (0.4)	
Pulseless Ventricular Fibrillation		23 (7.8)	8 (14.3)	15 (6.2)	
Asystole		155 (52.4)	4 (7.1)	151 (62.9)	
Normal Sinus Rhythm / no CPR		30 (10.1)	29 (51.8)	1 (0.4)	
Unknown		25 (8.4)	10 (17.9)	15 (6.2)	
No measures (obviously dead)		17 (5.7)	0 (0.0)	17 (7.1)	< 0.001
**Defibrillation**	296	37 (12.5)	10 (17.9)	27 (11.2)	0.178
**Number of shocks**	35	1.0 [1.0; 3.0]	1.0 [1.0; 1.0]	1.0 [1.0; 3.0]	0.243
Adrenaline	296	197 (66.6)	10 (17.9)	187 (77.9)	< 0.001
**Ongoing CPR during transport**	296	61 (20.6)	4 (7.1)	57 (23.8)	0.006
**Duration of CPR, minutes**	251	24.0 [8.0; 41.0]	0.0 [0.0; 10.0]	30.0 [15.0; 45.0]	< 0.001
Duration of bystander BLS, minutes	185	5.0 [0.0; 10.0]	2.0 [1.0; 5.0]	7.0 [0.0; 12.0]	0.066

Abbreviations: ALS = Advanced Life Support, BLS = Basic Life Support, CPR = Cardiopulmonary Resuscitation

Depending on normality testing (Shapiro Wilk) median [Q1;Q2] respectively mean (SD) are shown for continuous variables, *p*-values obtained by Wilcoxon rank sum test or unpaired T-test. Categorical variables are shown with number (%) in each category, *p*-values obtained by chi-squared test

**Fig. 2:**
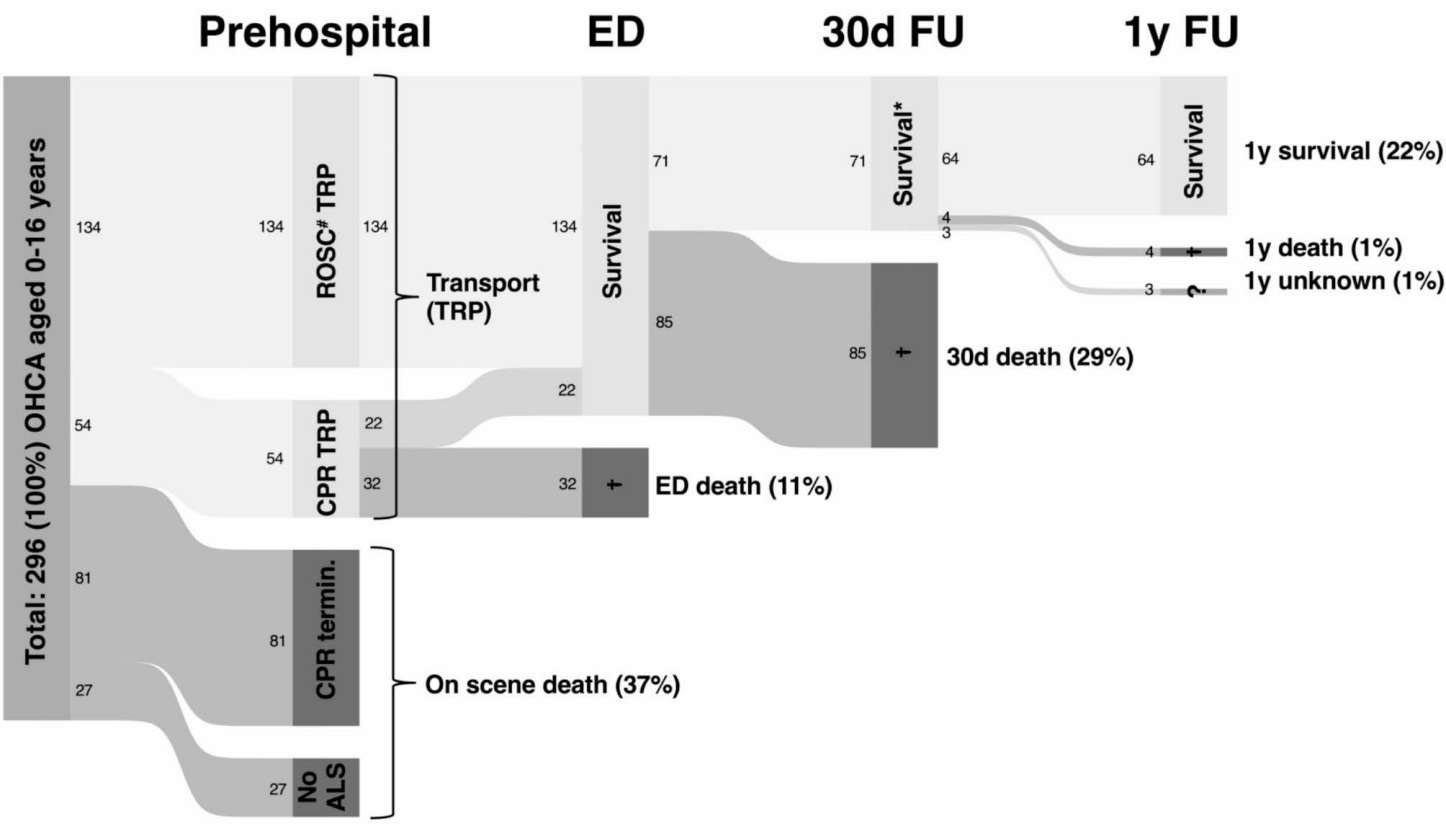
Sankey diagram showing survival after cardiac arrest. ^#^ 25% (n = 33) of ROSC patients had ROSC already achieved on HEMS arrival. * 80% (n = 56) of the 30d survivors with favourable neurological outcome (CPC 1/2). **Abbreviations**: ALS: advanced life support, CPR: cardiopulmonary resuscitation, ED: emergency department, FU: follow-up, OHCA: out-of-hospital cardiac arrest, ROSC: return of spontaneous circulation, TRP: transport, d: days, y: years, †: death, ?: unknown

The outcome data adjusted for favourable outcomes are presented in Table 2. Return of spontaneous circulation (ROSC) on scene was found in 45.3% (n = 134) of cases. Most ROSC was achieved by professional emergency medical services: 17.2% (n = 51) by ground-based emergency medical services and 16.9% (n = 50) by Rega-HEMS. ROSC was achieved by bystander CPR in 7.1% (n = 21) and in 4.1% (n = 12) by first responders BLS. Patient survival rates following cardiac arrest are displayed in Fig. 2. More than one-third (37.2%, n = 110) died on-scene, while the remaining two-thirds (62.8%, n = 186) were transported to an emergency department. At 30 days following the OHCA, 24.1% (n = 71) of patients were still alive, with a favourable neurological outcome being recorded in 18.9% (n = 56) patients: CPC 1 n = 46, 15.5%, and CPC 2 n = 10, 3.4%, which represent the majority (78.8%, n = 56) of the survivors. One year after the OHCA, 95.8% (n = 68) of 30-day survivors were assessable; only 4.2% (n = 3) had been lost to follow-up. Most of these (94.1%, n = 64) remained alive. Additional patient data are presented in the Supplement Figure S1 and Supplement Table S2.

**Table 2:** Outcomes of all cases and adjusted to 30-day favourable neurological outcome according to cerebral performance categories (CPC) 1 & 2

Variable	Total	30-day CPC 1 & 2		
	Total (n = 296)	Yes (n = 56)	No (n = 240)	*p*-value
Mission purpose				
No CPR started	27 (9.1)	0 (0.0)	27 (11.2)	
ROSC already achieved	33 (11.1)	33 (58.9)	0 (0.0)	
Termination of resuscitation attempt < 5 min after arrival	2 (0.7)	0 (0.0)	2 (0.8)	
Termination of resuscitation attempt > 5 min after arrival	79 (26.7)	0 (0.0)	79 (32.9)	
ALS and transport with pre-hospital ROSC	101 (34.1)	21 (37.5)	80 (33.3)	
ALS and transport without pre-hospital ROSC	54 (18.2)	2 (3.6)	52 (21.7)	< 0.001
Pre-hospital return of spontaneous circulation (ROSC)				
Yes	134 (45.3)	54 (96.4)	80 (33.3)	
No	162 (54.7)	2 (3.6)	160 (66.7)	< 0.001
ROSC achieved by				
Bystander	21 (7.1)	21 (37.5)	0 (0.0)	
First responder	12 (4.1)	12 (21.4)	0 (0.0.)	
Ground-based emergency medical system team	51 (17.2)	12 (21.4)	39 (16.2)	
Helicopter emergency medical system team	50 (16.9)	9 (16.1)	41 (17.1)	
After transportation (in-hospital)	24 (8.1)	2 (3.6)	22 (9.2)	
No ROSC at any time point	138 (46.6)	0 (0.0)	138 (57.5)	< 0.001
On-scene				
Survival	186 (62.8)	56 (100.0)	130 (54.2)	
Death	110 (37.2)	0 (0.0)	110 (45.8)	< 0.001
Emergency department				
Survival	156 (52.7)	56 (100.0)	100 (41.7)	
Death	140 (47.3)	0 (0.0)	140 (58.3)	< 0.001
30-days				
Survival	71 (24.0)	56 (100.0)	15 (6.2)	
Death	225 (76.0)	0 (0.0)	225 (93.8)	< 0.001
1-year				
Unknown	3 (1.0)	1 (1.8)	2 (0.8)	
Death	229 (77.4)	0 (0.0)	229 (95.4)	
Survival	64 (21.6)	55 (98.2)	9 (3.8)	< 0.001

Data presented in n (%)

Abbreviations: ALS = Advanced Life Support, CPR = Cardiopulmonary Resuscitation, ROSC = Return of Spontaneous Circulation

### Factors associated with favourable neurological outcome

Table 3 summarises the unadjusted univariable odds ratios for factors associated with a 30-day favourable neurological outcome, while Table 4 presents the results of the multivariable analysis. The most significant factors associated with favourable neurological outcomes were non-traumatic aetiology of the cardiac arrest (OR 11.07; 95%CI 2.38–51.42; *p* = 0.002) and initiating early bystander CPR (OR 10.34; 95%CI 2.29–46.72; *p* = 0.002). Factors associated with an increased likelihood of an unfavourable neurological outcome were: asystole as first rhythm (OR 0.12; 95%CI 0.04–0.39; *p* < 0.001), administration of adrenaline (OR 0.14; 95%CI 0.05–0.39; *p* < 0.001) and ongoing CPR upon HEMS team arrival (OR 0.17; 95%CI 0.04–0.65; *p* = 0.010).

**Table 3:** Univariable analysis for the 30-day favourable neurological outcome according to cerebral performance category (CPC) 1 & 2 of all cases (n = 296)

Variable	Odds ratio	(95% CI)	*p*-value
Weekend mission	1.67	(0.90; 3.10)	0.106
Season			
Winter	1.00	(baseline)	
Spring	0.89	(0.38; 2.07)	0.792
Summer	1.20	(0.54; 2.66)	0.655
Fall	0.71	(0.27; 1.89)	0.492
Helicopter hoist operation	0.29	(0.04; 2.28)	0.241
Remote location	1.06	(0.49; 2.26)	0.890
Age in years	0.95	(0.90; 1.01)	0.092
Age group			
< 1 years	1.00	(baseline)	
1–6 years	1.33	(0.61; 2.92)	0.474
7–16 years	0.64	(0.28; 1.47)	0.291
Sex			
Male	1.00	(baseline)	
Female	1.14	(0.63; 2.06)	0.662
Pre-existing co-morbidities			
Cardiovascular	1.15	(0.37; 3.62)	0.806
Respiratory	0.42	(0.05; 3.34)	0.411
Premature birth	1.44	(0.15; 14.07)	0.756
Neurological	1.64	(0.42; 6.40)	0.475
Syndrome	0.71	(0.08; 6.01)	0.753
Diabetes	-		
Dispatcher-identified cardiac arrest	1.43	(0.79; 2.56)	0.236
Dispatcher-assisted CPR	0.94	(0.41; 2.16)	0.892
Witnessed cardiac arrest	1.79	(1.00; 3.23)	0.051
Bystander CPR	8.52	(2.98; 24.32)	**< 0.001**
ALS before HEMS arrival	0.74	(0.40; 1.37)	0.339
Ongoing chest compressions at HEMS arrival	0.14	(0.07; 0.27)	**< 0.001**
Use of mechanical CPR device	-		
Resuscitation delay (> 2 min)	0.18	(0.09; 0.36)	**< 0.001**
Initial rhythm			
Asystole	0.03	(0.01; 0.10)	**< 0.001**
Pulseless electrical activity (PEA)	0.49	(0.18; 1.31)	0.154
Shockable rhythm	1.29	(0.45; 3.65)	0.636
Number of shocks	0.69	(0.34; 1.41)	0.311
Adrenaline	0.06	(0.03; 0.13)	**< 0.001**
Tracheal tube	0.26	(0.14; 0.48)	**< 0.001**
Transport under ongoing CPR	0.25	(0.09; 0.71)	**0.010**
Traumatic aetiology	0.09	(0.03; 0.29)	**< 0.001**
Non-traumatic aetiology	11.37	(3.45; 37.45)	**< 0.001**

Data presented in odds ratios and corresponding 95% confidence interval (95% CI).

Abbreviations: ALS = Advanced Life Support, CPR = Cardiopulmonary Resuscitation, HEMS = Helicopter Emergency Medical Service, PEA = Pulseless Electrical Activity

**Table 4:** Multivariable analysis for the 30-day favourable neurological outcome according to cerebral performance category (CPC 1 & 2) for all patients, excluding both patients without any resuscitation attempt (subgroup 1) and patients without any resuscitation attempt or ROSC already achieved (subgroup 2)

Variable	All patients (n = 296)			Subgroup 1 (n = 269)			Subgroup 2 (n = 236)		
	OR	(95% CI)	*p*-value	OR	(95% CI)	*p*-value	OR	(95% CI)	*p*-value
Non-traumatic cardiac arrest	11.07	(2.38; 51.42)	**0.002**	4.11	(0.89; 18.96)	0.070	3.59	(0.76; 17.02)	0.107
Asystole (first rhythm)	0.12	(0.04; 0.39)	**< 0.001**	0.11	(0.03; 0.37)	**< 0.001**	0.15	(0.05; 0.49)	**0.002**
Bystander CPR	10.34	(2.29; 46.72)	**0.002**	2.22	(0.58; 8.55)	0.247	1.90	(0.50; 7.31)	0.349
Ongoing CPR on HEMS arrival	0.17	(0.04; 0.65)	**0.010**	0.03	(0.00; 0.29)	**0.003**	0.11	(0.00; 3.25)	0.204
Adrenaline	0.14	(0.05; 0.39)	**< 0.001**	0.13	(0.05; 0.34)	**< 0.001**	0.21	(0.07; 0.59)	**0.003**
	AUROC = 0.924.			AUROC = 0.939.			AUROC = 0.861.		

Data given in Odds Ratios (OR) with corresponding 95% confidence interval (95% CI)

An area under the receiver operating characteristic curve (AUROC) greater than 0.8 indicates an excellent model fit

Abbreviations: BLS = Basic Life Support, CPR = Cardiopulmonary Resuscitation, HEMS = Helicopter Emergency Medical System, ROSC = Return of Spontaneous Circulation

Bold p-values are significant

In the analysis of subgroup 1 (n = 269), excluding patients without any resuscitation attempt (bystander CPR, BLS or ALS) (n = 27), asystole (OR 0.11; 95%CI 0.03–0.37; *p* < 0.001), administration of adrenaline (OR 0.13; 95%CI 0.05–0.34; *p* < 0.001) and ongoing CPR upon the arrival of the HEMS team (OR 0.03; 95%CI 0.00-0.29; *p* = 0.003) remained statistically significant factors associated with an unfavourable neurological outcome. In the analysis of subgroup 2 (n = 236), excluding patients without any resuscitation attempt (bystander CPR, BLS or ALS) (n = 27) or ROSC already achieved (n = 33), only asystole (OR 0.15; 95%CI 0.05–0.49; *p* = 0.002) and administration of adrenaline (OR 0.21; 95%CI 0.07–0.59; *p* = 0.003) remained as factors with a statistically significant association with unfavourable neurological outcomes.

## Discussion

This 11-year retrospective observational study identified favourable neurological outcome in 18.9% of paediatric patients 30 days after OHCA. BLS and non-traumatic aetiology for the cardiac arrest were associated with favourable neurological outcome at 30 days, while initial asystole and administration of adrenaline were associated with an unfavourable neurological outcome.

### Importance of immediate bystander CPR and first responder BLS

Despite potential advances in managing paediatric OHCA, traditional survival rates remain low. Gelberg et al. [13] observed in paediatric OHCA patients (up to 21 years of age) an increase from 6.2 to 14% in overall 30-day neurological favourable survival from 1990 to 2012. Similarly, Law et al. [14] investigated patients admitted to hospital with out-of-hospital cardiac arrest with only 28.3% bystander CPR and found a survival rate of 20.8%, with only 13.2% having a favourable neurological outcome. In contrast, the survival rate was much higher in our cohort, even though we included patients who were declared dead on-scene. Differences in the approaches of local emergency medical systems might explain this discrepancy.

In our population, two-thirds of the patients were treated with immediate bystander CPR, resulting in 7.1% attaining ROSC before the arrival of the Rega-HEMS team. Additionally, first responder BLS resulted in 4.1% attaining ROSC. All these patients survived with 30-day favourable neurological outcome. The importance of immediate CPR for paediatric OHCA was clearly described earlier [13, 15–18]. A Japanese OHCA registry analysis from 2005 included 5,170 children under the age of 18 years and found an adjusted odds ratio of 2.59 (95% CI 1.81–3.71) for a favourable neurological outcome with BLS [18]. The fact that the odds ratio in our cohort was four times higher may be a result of improvements in resuscitation in the last 15 years. This includes broad BLS education for laypersons that nowadays also includes teaching CPR to children, [19] the implementation of dispatcher-assisted CPR [20] and the introduction of nationwide first-responder systems [21, 22].

Nonetheless, closing the gap for the one-third of patients not treated with immediate CPR may be challenging, as some patients need to be rescued from remote areas that cannot be directly accessed by foot. A registry analysis underlined that early advanced life support is crucial for favourable neurological survival in adults [23]. These findings may hold true for the paediatric population as well, thus justifying possible interventions by HEMS that provide access to almost all areas.

That said, most resuscitation-awareness campaigns for laypersons focus on adult BLS education [24]. Given the entirely different physiology and aetiology of OHCA in children as compared to adults, of which higher oxygen consumption and hypoxemia is a leading cause, these differences must be considered and incorporated into such campaigns and highlighted in future resuscitation recommendations.

### Traumatic cardiac arrest in children

The resuscitation of children, especially after major trauma, represents a substantial challenge for laypersons, as first responders, and for professional medical rescuers, because such cases are rare. Rescuers face significant emotional burdens and the rescue teams are often not as well trained in managing children as victims, as compared to adult patients. The proportion of paediatric emergencies within the total case volume of HEMS is about 8%.^25^ Traumatic cardiac arrest might be more challenging for dispatchers to identify because it is a rare condition, and the patient’s unresponsiveness might be misinterpreted due to traumatic brain injury. This might lead to less dispatcher-assisted CPR [26]. The invasive measures necessary to correct reversible trauma-related causes (e.g. relief of tension pneumothorax or pericardial effusion) are challenging and rarely performed, even more so in children [25–27]. Trauma was the second-most-common cause of out-of-hospital cardiac arrest in children in our study, and boys were more likely to be victims of major trauma (higher risk-taking and older age), which aligns with other reports in the prehospital care of children [4]. The difficulty of access by foot or the need for a helicopter hoist operation often slows down the procedure, resulting in delayed resuscitation efforts [25].

Even in children, unrecognised reversible causes remain a problem in cardiac arrest after major trauma. Ismail et al. demonstrated that 113 (24%) of 472 paediatric trauma patients required chest decompression during treatment, indicating that severe chest trauma is not rare in children [28]. Consistent with this impression, a post-mortem CT study showed that 70 (96%) of 73 children who died had at least one concomitant chest injury, of which the medical team was, in many cases, unaware [29]. Thus, a patient with undiagnosed and untreated severe thoracic injury may not enjoy the benefit of the best resuscitative efforts.

Moreover, in our study, children have a higher survival rate after trauma than adults, but they also usually have worse neurological outcomes than adult trauma patients [30]. In a retrospective study from Japan of 582 children who underwent CPR after trauma, a resuscitation time of more than 15 min was associated with poor neurological outcomes in children [31]. These findings are consistent with our results.

### Topics to be addressed in the future

There is an ongoing discussion of the advantages of tracheal intubation in ALS. In our univariable analysis, tracheal intubation was associated with reduced odds for favourable neurological outcome at 30 days, but this no longer held true in the multivariable analysis. This may be explained by the high proportion of patients with favourable neurological survival with ROSC after BLS who were spontaneously breathing without needing airway management in post-resuscitation care. In contrast, a German registry analysis including over 1,700 paediatric OHCA cases found higher odds ratios for ROSC with advanced airway management. Unfortunately, their data lack 30-day survival and neurological outcomes. Compared to our data, the German Registry analyses around 10% of traumatic cardiac arrests and the authors excluded patients declared deceased on arrival, which might explain different findings [6]. A French registry analysis, including over 1,500 paediatric OHCA patients, comparing tracheal intubation with supraglottic airway didn’t find higher odds ratios in their propensity-scored analysis for the outcome ROSC. Furthermore, they found poorer odds ratios for 30-day survival and neurological outcome for patients with tracheal intubation [32].

The multivariable model found an association between adrenaline and reduced odds for 30-day unfavourable neurological outcomes. This association might be explained by resuscitation time bias, which leads to more interventions the longer the duration of CPR is. This hypothesis might be underlined by the finding that a shorter duration of CPR was significantly associated with favourable neurological outcomes in our univariable analysis. Unfortunately, we couldn’t demonstrate this in the multivariable analysis due to too many missing for the duration of CPR.

In our cohort, on-scene time was significantly shorter for patients with favourable 30-day survival. However, this might be explained by the helicopter take-off time-stamp used. In the event of a declaration of death on-scene, legal documents must be filled in by the responsible HEMS physician on-scene, prior to take-off.

Early damage-control surgery may be a crucial factor in survival, especially for patients with traumatic cardiac arrest who are suffering from non-compressible haemorrhage. This might justify the use of scoop-and-run tactics and transport under ongoing CPR, without any delaying interventions being performed on-scene, such as advanced airway management. These interventions could instead be performed in the helicopter on the way to hospital, thus saving precious time.

### Limitations

Due to the retrospective design of our study, one limitation is that missing variables cannot be reproduced, and we did not impute such data. The missions were classified by the treating HEMS physician on-scene according to the NACA score, which included some patients with ROSC upon arrival of the HEMS team. Asystole as the first rhythm and the administration of adrenaline were associated with unfavourable neurological outcome. However, there is a clear association between these factors, as, following the guidelines, all non-shockable (i.e. asystole and PEA) rhythms are treated with adrenaline. We were unable to overcome the potential resuscitation time bias due to missing data. We report a large cohort of traumatic cardiac arrests, which might be caused by selection bias: HEMS can easily reach recreational areas like lakes, rivers, and alpine regions, but landing with a helicopter in cities or crowded areas can be challenging or impossible. Thus, our data might not represent the entire Swiss emergency medical services.

## Conclusions

In this study, 18.9% of paediatric OHCA patients survived with a favourable neurologic outcome 30 days after treatment by the Swiss helicopter emergency medical service. Immediate bystander cardiopulmonary resuscitation and non-traumatic OHCA aetiology were the factors most strongly associated with a favourable neurological outcome. These results underline the importance of effective bystander and first-responder rescue as the foundation for subsequent professional treatment of children in cardiac arrest.

### Electronic supplementary material

Below is the link to the electronic supplementary material.

**Supplementary Material 1**: **Supplement figure S1** Patient characteristics and outcomes according to the Utstein-style flowchart.

**Supplementary Material 2**: **Supplement Table S2** Detailed baseline characteristics of all cases and adjusted to 30-day favourable neurological outcome defined as cerebral performance categories (CPC) 1 and 2. Data presented in n (%).

## Data Availability

The presented data in the manuscript is available from the authors with a reasonable request and after permission of the responsible ethical committee due to Swiss law.

## Abbreviations

ALS: Advanced Life Support
BLS: Basic Life Support
CPC: Cerebral Performance Category
HEMS: Helicopter Emergency Medical Service
ICD: International Classification of Diseases
NACA: National Advisory Committee for Aeronautics
OHCA: Out-of-Hospital Cardiac Arrest
