# Supplementary material for: Favourable neurological outcome following paediatric out-of-hospital cardiac arrest: a retrospective observational study
Source: Scand J Trauma Resusc Emerg Med. 2023 Dec 21;31:106. doi: 10.1186/s13049-023-01165-y (PMC10734091; doi:10.1186/s13049-023-01165-y)
Supplement: Supplementary file 1 — Supplementary Material 1: Supplement figure S1 Patient characteristics and outcomes according to the Utstein-style flowchart. [file 13049_2023_1165_MOESM1_ESM.docx]

**Supplement Figure S1.** Patient characteristics and outcomes according to the Utstein-style flowchart
