# Supplementary material for: Favourable neurological outcome following paediatric out-of-hospital cardiac arrest: a retrospective observational study
Source: Scand J Trauma Resusc Emerg Med. 2023 Dec 21;31:106. doi: 10.1186/s13049-023-01165-y (PMC10734091; doi:10.1186/s13049-023-01165-y)
Supplement: Supplementary file 2 — Supplementary Material 2: Supplement Table S2 Detailed baseline characteristics of all cases and adjusted to 30-day favourable neurological outcome defined as cerebral performance categories (CPC) 1 and 2. Data presented in n (%). [file 13049_2023_1165_MOESM2_ESM.docx]

### Supplement Table S2. Detailed baseline characteristics of all cases and adjusted to 30-day favourable neurological outcome defined as cerebral performance categories (CPC) 1 and 2. Data presented in n (%)

|  | Total | 30-day CPC 1 & 2 | |  |
| --- | --- | --- | --- | --- |
|  | Total  (n=296) | Yes (n=56) | No (n=240) | P-value |
| Mission day |  |  |  |  |
| Sunday | 40 (13.5) | 9 (16.1) | 31 (12.9) |  |
| Monday | 41 (13.9) | 7 (12.5) | 34 (14.2) |  |
| Tuesday | 41 (13.9) | 8 (14.3) | 33 (13.8) |  |
| Wednesday | 46 (15.5) | 9 (16.1) | 37 (15.4) |  |
| Thursday | 44 (14.9) | 8 (14.3) | 36 (15.0) |  |
| Friday | 44 (14.9) | 4 (7.1) | 40 (16.7) |  |
| Saturday | 40 (13.5) | 11 (19.6) | 29 (12.1) | 0.524 |
| Night mission (20:00–07:59) |  |  |  |  |
| No | 251 (84.8) | 55 (98.2) | 196 (81.7) |  |
| Yes | 45 (15.2) | 1 (1.8) | 44 (18.3) | 0.002 |
| Activity (main) |  |  |  |  |
| Transport | 43 (14.5) | 2 (3.6) | 41 (17.1) |  |
| Flying | 2 (0.7) | 0 (0.0) | 2 (0.8) |  |
| Water sports | 38 (12.8) | 19 (33.9) | 19 (7.9) |  |
| Summer sports | 5 (1.7) | 4 (7.1) | 1 (0.4) |  |
| Winter sports | 11 (3.7) | 2 (3.6) | 9 (3.8) |  |
| Mountain sports | 10 (3.4) | 1 (1.8) | 9 (3.8) |  |
| Work | 5 (1.7) | 0 (0.0) | 5 (2.1) |  |
| Other | 182 (61.5) | 28 (50.0) | 154 (64.2) | <0.001 |
| Activity (detail) |  |  |  |  |
| Car/bus | 1 (0.3) | 0 (0.0) | 1 (0.4) |  |
| Car | 8 (2.7) | 0 (0.0) | 8 (3.3) |  |
| Motorbike | 4 (1.4) | 0 (0.0) | 4 (1.7) |  |
| Moped | 2 (0.8) | 0 (0.0) | 2 (0.8) |  |
| Bicycle | 7 (2.4) | 1 (1.8) | 6 (2.5) |  |
| Pedestrian | 14 (4.7) | 1 (1.8) | 13 (15.4) |  |
| Chairlift/ski-lift | 1 (0.3) | 0 (0.0) | 1 (0.4) |  |
| Agricultural and forestry vehicles | 2 (0.7) | 0 (0.0) | 2 (0.8) |  |
| Other means of transport | 4 (1.4) | 0 (0.0) | 4 (1.7) |  |
| Motorised aircraft | 2 (0.7) | 0 (0.0) | 2 (0.8) |  |
| Swimming | 37 (12.5) | 19 (33.9) | 18 (7.5) |  |
| Canoe/kayak | 1 (0.2) | 0 (0.0) | 1 (0.4) |  |
| Hiking | 1 (0.3) | 1 (1.8) | 0 (0.0) |  |
| Mountain biking | 1 (0.3) | 0 (0.0) | 1 (0.4) |  |
| Other summer sport | 3 (1.0) | 3 (5.4) | 0 (0.0) |  |
| Skiing/snowboarding | 9 (3.0) | 2 (3.6) | 7 (2.9) |  |
| Other winter sport | 2 (0.7) | 0 (0.0) | 2 (0.8) |  |
| Hiking (alpine) | 7 (2.4) | 1 (1.8) | 6 (2.5) |  |
| Alpine touring | 1 (0.3) | 0 (0.0) | 1 (0.4) |  |
| Climbing | 2 (0.7) | 0 (0.0) | 2 (0.8) |  |
| In agriculture | 3 (1.0) | 0 (0.0) | 3 (1.2) |  |
| In forestry | 2 (0.7) | 0 (0.0) | 2 (0.8) |  |
| In and around the house | 145 (49.0) | 22 (39.3) | 123 (51.2) |  |
| Horse-riding | 2 (0.7) | 0 (0.0) | 2 (0.8) |  |
| Relocation (secondary deployment) | 2 (0.7) | 0 (0.0) | 2 (0.8) |  |
| No activity or unknown | 33 (11.1) | 6 (10.7) | 27 (11.2) | 0.001 |
| Aetiology detail |  |  |  |  |
| *Non-traumatic* |  |  |  |  |
| Asphyxia – drowning | 64 (21.6) | 23 (41.) | 41 (17.1) |  |
| Asphyxia – aspiration | 25 (8.4) | 4 (7.1) | 21 (8.8) |  |
| Asphyxia – strangulation | 13 (4.4) | 3 (5.4) | 10 (4.2) |  |
| Asphyxia – burial | 6 (2.0) | 2 (3.6) | 4 (1.7) |  |
| Other pulmonary | 16 (5.4) | 3 (5.4) | 13 (5.4) |  |
| Cardiac | 23 (7.8) | 9 (16.1) | 14 (5.8) |  |
| Sudden infant death syndrome (SIDS) | 23 (7.8) | 0 (0.0) | 23 (9.6) |  |
| Anaphylactic | 2 (0.7) | 0 (0.0) | 2 (0.8) |  |
| Intoxication | 1 (0.3) | 1 (1.8) | 0 (0.0) |  |
| Non-traumatic, reason unknown | 17 (5.7) | 5 (8.9) | 12 (5.0) |  |
| Other non-traumatic | 9 (3.0) | 3 (5.4) | 6 (2.5) |  |
| *Traumatic* |  |  |  |  |
| Traffic accident | 45 (15.2) | 0 (0.0) | 45 (18.8) |  |
| Fall from a height | 25 (8.4) | 3 (5.4) | 22 (9.2) |  |
| Burning/scalding | 3 (1.0) | 0 (0.0) | 3 (1.2) |  |
| Other traumatic | 24 (8.1) | 0 (0.0) | 24 (10.0) | <0.001 |
| First rhythm |  |  |  |  |
| Asystole | 155 (52.4) | 4 (7.1) | 151 (62.9) |  |
| Pulseless Electrical Activity | 45 (15.2) | 5 (8.9) | 40 (16.7) |  |
| Pulseless Ventricular Tachycardia | 1 (0.3) | 0 (0.0) | 1 (0.4) |  |
| Pulseless Ventricular Fibrillation | 23 (7.8) | 8 (14.3) | 15 (6.2) |  |
| Normal Sinus rhythm / ROSC | 30 (10.1) | 29 (51.8) | 1 (0.4) |  |
| Unknown | 25 (8.4) | 10 (17.9) | 15 (6.2) |  |
| No measures taken (obviously dead) | 17 (5.7) | 0 (0.0) | 17 (7.1) | <0.001 |
| Injuries |  |  |  |  |
| Traumatic brain injury | 72 (24.3) | 3 (5.4) | 69 (28.7) | <0.001 |
| Chest trauma | 31 (10.5) | 0 (0.0) | 31 (12.9) | 0.004 |
| Abdominal trauma | 20 (6.8) | 0 (0.0) | 20 (8.3) | 0.025 |
| Pelvic trauma | 6 (2.0) | 0 (0.0) | 6 (2.5) | 0.232 |
| Upper extremity trauma | 1 (0.3) | 0 (0.0) | 1 (0.4) | 0.628 |
| Lower extremity trauma | 13 (4.4) | 0 (0.0) | 13 (5.4) | 0.075 |

Categorical variables are shown with numbers (%) in each category, p-values obtained by chi-squared test.
